# Chemical quantification and antioxidant assay of four active components in *Ficus hirta* root using UPLC-PAD-MS fingerprinting combined with cluster analysis

**DOI:** 10.1186/1752-153X-7-115

**Published:** 2013-07-08

**Authors:** Tao Yi, Qilei Chen, Xicheng He, Suiwai So, Yuenling Lo, Lanlan Fan, Jun Xu, Yina Tang, Jianye Zhang, Zhongzhen Zhao, Hubiao Chen

**Affiliations:** 1 School of Chinese Medicine, Hong Kong Baptist University, Hong Kong Special Administrative Region, Hong Kong, P. R. China; 2 Guangxi Botanical Garden of Medicinal Plant, Nanning, China

**Keywords:** *Ficus hirta*, UPLC-PAD-MS, Chemical quantification, Fingerprinting, Antioxidant capacity

## Abstract

**Background:**

Root of *Ficus hirta* (RFH) is widely consumed in China as a plant-derived popular food. However, contents of the active constituents of RFH are unknown, and the chemical as well as bioactive properties of RFH may be affected by growing area. In order to ensure the standard efficacy of health products made with RFH, its active constituents should firstly be determined and, secondly, a means of assessing samples for their contents of these constituents is needed.

**Results:**

Four active components, including two coumarins, namely psoralen and bergapten, and two flavonoids, namely luteolin and apigenin, in twenty RFH samples were quantified using a new ultra performance liquid chromatography coupled with photodiode array detector and mass spectrometry (UPLC-PAD-MS) method, and the content level in descending order was psoralen > bergapten > luteolin > apigenin. Chromatographic fingerprint similarity evaluation and cluster analysis were used to assess geographical origin of RFH, and the results revealed a high level of similarity for the tested RFH samples obtained from Hainan, Guangdong, Guangxi provinces and Hong Kong. 2, 2-Diphenyl-1-picrylhydrazyl (DPPH) radical scavenging assay was conducted to evaluate the antioxidant potencies of the four components, and the results clearly demonstrated that luteolin was most effective; apigenin exhibited a moderate potency, whereas psoralen and bergapten possessed little effect against free radical reactions. Structure-activity relationship of the components was elucidated, and the 3′-hydroxyl group of luteolin was found to be directly responsible for its antioxidant activity.

**Conclusion:**

The present UPLC-PAD-MS method and DPPH radical scavenging assay performed well for the purpose of constituent quantification and antioxidant assay. Global profiles were highly similar for RFH samples from different origins. Both the coumarins and flavonoids were involved in the health benefit of RFH.

## Background

The root of *Ficus hirta* (RFH) is a plant-derived food and has been widely consumed in China [1,2]. Beside direct consumption, a number of health products have been developed from RFH, such as beverages, teas, and wines, which are selling briskly. Recent studies have also revealed that RFH has immune regulatory [3], antibacterial [4], anti-inflammatory and analgesic effects [5], thus RFH also has potential values in human healthcare [6].

Our previous study reported that RFH soup has a clear protective effect against dimethylformamide- and cocaine-induced acute liver injury in mice via inhibition of free radical reactions [7,8]. We also found that RFH contains potentially active constituents, such as psoralen, bergapten, luteolin and apigenin [7]. However, contents of the active constituents of RFH are unknown, and their radical scavenging effects are not compared. Moreover, there is more than one RFH-growing area in southern China, and the chemical and bioactive properties of RFH may be affected by growing area. In order to ensure the standard efficacy of health products made with RFH, its active constituents should firstly be determined and, secondly, a means of assessing samples for their contents of these constituents is needed.

Recently, ultra performance liquid chromatography has been coupled with photodiode array detector and mass spectrometry (UPLC-PAD-MS) to create a highly specific, precise, and accurate method that is readily applicable to the quality control of botanical products [9-11]. Chromatography fingerprinting coupled with chemometrics has also become one of the most frequently applied approaches in evaluation of chemical profiles of botanical products [12-14]. 2, 2-Diphenyl-1-picrylhydrazyl (DPPH) radical scavenging, with its advantages of simplicity and efficiency, is a valuable tool for evaluating the antioxidant potency of health products [15-18]. These are promising approaches to clarifying our unsolved problem.

In the present study, a new UPLC-PAD-MS method for the qualitative and quantitative analysis of RFH obtained from five regions has been developed and validated. Four active components were targeted; these were psoralen, bergapten, luteolin and apigenin. Chromatographic fingerprint similarity evaluation and cluster analysis were used to assess geographical origin of RFH, and the results revealed a high level of similarity for the tested RFH samples. DPPH radical scavenging assay was conducted to compare the antioxidant potencies of these four components. Luteolin exhibited the strongest activity in the antioxidant assay, and its 3′-hydroxyl group was found to be directly responsible for the antioxidant activity based on a structure-activity relationship analysis.

## Experimental

### Reagents

Analytical grade methanol (Labscan, Bangkok, Thailand) was used for preparation of standards and sample extraction. Chromatographic grade acetonitrile (Labscan, Bangkok, Thailand), chromatographic grade formic acid (Fluka, Buchs, Switzerland) and deionized water obtained from a Milli-Q water purification system (Millipore, Bedford, MA, USA) were used for preparation of the mobile phase. Analytical grade ethanol (Merck, Darmstadt, Germany) was used as the solvent in the antioxidant assay.

The standard compounds of luteolin and apigenin were purchased from Phytomarker Co. Ltd. (Tianjin, China). Psoralen, bergapten and 2, 2-diphenyl-1-picrylhydrazyl (DPPH) were purchased from Sigma Chemical Co. (St. Louis, MO, USA). Scopoletin (Phytomarker Co. Ltd, Tianjin, China) and quercetin (National Institute for the Control of Pharmaceutical and Biological Products, Beijing, China) were used as positive controls in the antioxidant assay.

## Materials

A total of 20 fresh samples of root of *Ficus hirta* (RFH) were collected as plants from four regions in China, namely, Hainan province (samples 1-4), Guangdong province (samples 5-8), Guangxi province (samples 9-11), and Hong Kong (samples 18-20), and as commercial products purchased from stores in Hong Kong (samples 12-17). All samples were authenticated by Dr. Chen Hubiao (School of Chinese Medicine, Hong Kong Baptist University), and the corresponding voucher specimens were deposited in our laboratory.

### Sample preparation

For the chemical quantification, each RFH sample (1.0 g) was accurately weighed and extracted with 10.0 mL of 80% methanol by sonication at room temperature for 30 min. The extraction was repeated two times, and the total extracts were combined in a 25 mL volumetric flask. 80% methanol was added to make the volume up to 25.0 mL. Three replicates of each sample were prepared and filtered through an Alltech (Beerfield, IL, USA) syringe filter (0.2 μm) before UPLC analysis. The four reference compounds were accurately weighed and dissolved in 80% methanol to produce standard stock solutions. Each stock solution was diluted to yield a series of standard solutions in the concentration range of 0.5-10.0 mg/L for luteolin, psoralen and bergapten, and 0.1-2.0 mg/L for apigenin.

For the antioxidant assay, DPPH test solution of 0.1 mM was prepared by dissolving 19.72 mg DPPH in 500 mL ethanol; it was stored away from light. Stock solutions of luteolin, psoralen, apigenin, bergapten, scopoletin (positive control for coumarins) and quercetin (positive control for flavonoids) were prepared in ethanol and stored in the refrigerator. The working solutions were prepared by appropriate dilution of the stock solutions with ethanol, and the resulting concentration ranges were 0.01-0.15 mg/mL for psoralen, bergapten, apigenin and scopoletin, while 0.01-0.05 mg/mL for luteolin and quercetin.

### Analytical procedure

For the chemical quantification, a Waters Acquity™ ultra performance liquid chromatography (UPLC) system (Waters Corp., Milford, USA) coupled with a photodiode array detector (PAD) and a MicroTOF-Q mass spectrometry (Bruker Daltonics, Bremen, Germany) was used. Separation was performed on a VanGuard™ HSS C_18_ column (1.8 μm, 2.1 mm × 100 mm, Waters Corp.) at 40°C. The mobile phase consisted of 0.1% formic acid in water and 0.1% formic acid in acetonitrile using a gradient program of 3% in 0-2.5 min, 3-35% in 2.5-11 min, 35-85% in 11-21 min and 85-100% in 21-21.5 min. The injection volume of samples and standards was 3 μL and detection was performed at 270 nm. The conditions of MS analysis in the positive and negative ion mode were as follows: drying gas (nitrogen), flow rate, 8.0 mL/min; gas temperature, 200°C; scan range, 50-1000 m/z; capillary voltage, 4500 V; nebulizer press, 1.5 Bar.

For the antioxidant assay, an UV–vis spectrophotometer (Jasco V530, Japan Servo Co. Ltd., Japan) was used. Ethanol solutions (0.5 mL) of standards or positive control compounds were mixed with 1.5 mL DPPH ethanol solution, and the mixtures were kept from light in room temperature for 30 minutes. The absorption (A_1_) of each mixture was tested at a wavelength of 517 nm. A blank control with 0.5 mL ethanol and 1.5 mL DPPH ethanol solution was treated with the same above procedures to record its absorption (A_0_). Each test solution was repeated three times, and the average value was calculated as its DPPH free radical scavenging percentage, according to this formula: DPPH free radical scavenging (%) = ((A_0_ – A_1_)/A_0_) × 100.

## Results and discussion

### Optimization of the extraction and analysis conditions

The conditions of extraction method, solvents and times were optimized. Possible extraction methods were sonication, reflux and soxhlet extraction [19]; of these, sonication was found to be the easiest and most efficient. Compared to methanol, ethanol and their various concentrations of aqueous solution, extraction with 80% methanol produced the highest yield for the desired analytes. Comparative tests of various extraction times and cycles revealed that exhaustive extraction could be achieved when 0.1 g RFH sample powder was extracted with 10 mL 80% methanol by means of sonication for 0.5 h, twice.

**Figure 1:**
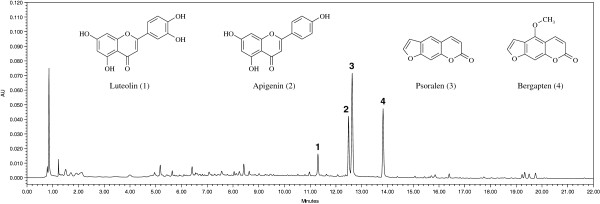
Typical UPLC chromatogram of RFH sample at 270 nm with the chemical structures of the identified peaks (peak 1, luteolin; peak 2, apigenin; peak 3, psoralen; peak 4, bergapten).

Chromatographic separations were assessed by eluting the RFH extract on a HSS C_18_ column with different mobile phase compositions, and it was shown that mobile phase consisting of acetonitrile and water gave the best separation at a lower column pressure. After comparing the chromatograms of the RFH samples recorded at wavelengths within 190–500 nm, it was found that 270 nm could best represent the profile of the analytes. The representative UPLC chromatograms are shown in Figure 1 and the UV absorption maximum for each analyte is listed in Table 1.

**Table 1:** MS and spectral data of the identified peaks in the UPLC chromatogram

**Peak no.**	**RT (min)**	**Components**	**Formula**	**MW**	**[*M *+ H]**^**+ **^**(m/z)**	**[*M *+ Na]**^**+ **^**(m/z)**	**[*M *- H]**^**- **^**(m/z)**	**λ **_**max **_**(nm)**
1	11.2	Luteolin	C_15_H_10_O_6_	286	287	309	285	253, 348
2	12.3	Apigenin	C_15_H_10_O_5_	270	271	293	269	267, 337
3	12.5	Psoralen	C_11_H_6_O_3_	186	187	209	-	246, 293
4	13.7	Bergapten	C_12_H_8_O_4_	216	217	239	-	268, 312

The mass spectrum was acquired by both positive and negative ion modes. Based on recorded m/z values, UV spectra and a comparison with standard compounds, four peaks were unambiguously identified as lutoelin (1), apigenin (2), psoralen (3) and bergapten (4). Their mass data is listed in Table 1.

The choice of solvent used in DPPH assay was compared in ethanol and methanol, and the results shown that ethanol has the ability to fully dissolve DPPH and analytes, and is not toxic to the environment [20]. Therefore, ethanol was recommended as the solvent for preparation of DPPH and analyte solutions. After scanning the DPPH in ethanol from 300–650 nm, the maximum absorption wavelength of 517 nm was chosen to monitor the absorption of the assay mixtures. The ratio of DPPH and analyte solution as well as the reaction time was further optimized, until the appropriated scavenging percentages were observed for the analytes.

### Method validation

Method validation parameters included linearity, reproducibility, precision and recovery. The 5-point calibration curves were constructed by plotting the peak area (mAU) of the analytes against the concentration (mg/L). The linear regression equation and correlation coefficient (*R*^*2*^) were y = 22069x – 3101 (*R*^*2*^ 0.9978) for luteolin, y = 28139x + 175 (*R*^*2*^ 0.9924) for apigenin, y = 12777x – 671 (*R*^*2*^ 1.0000) for psoralen and y = 27908x – 1739 (*R*^*2*^ 0.9999) for bergapten. Based on visual evaluation with a signal to noise ratio of about 3:1, the limit of detection (LOD) of the quantified constituents was found to be less than 1.5 ng. Satisfactory linearity and sensitivity for the analysis for the four analytes was obtained.

Method reproducibility was evaluated by five replicated analyses of RFH samples (*n* = 5). The relative standard deviation (RSD) values of the content of luteolin, apigenin, psoralen and bergapten were 3.30%, 4.58%, 0.89% and 0.47%, respectively. Method precision was investigated by repeatedly analyzing the same set of standard solution (*n* = 5), and the RSDs of calculated concentration were 0.79%, 0.92%, 0.80% and 0.62% for luteolin, apigenin, psoralen and bergapten, respectively. Recovery of the four components was determined by samples at different concentration levels using a mixture of standards with 100% of the quantified levels of components in five replicated RFH sample (*n* = 5). The average recovery of luteolin, apigenin, psoralen and bergapten were 97.68% (RSD 1.73%), 97.28% (RSD 1.81%), 97.96% (RSD 1.24%) and 96.24% (RSD 2.22%), respectively. The overall analytical procedure is accurate and reproducible and accommodates high throughput; it is suitable for chemical quantification of a large number of RFH samples.

### Chemical quantification of RFH

**Figure 2:**
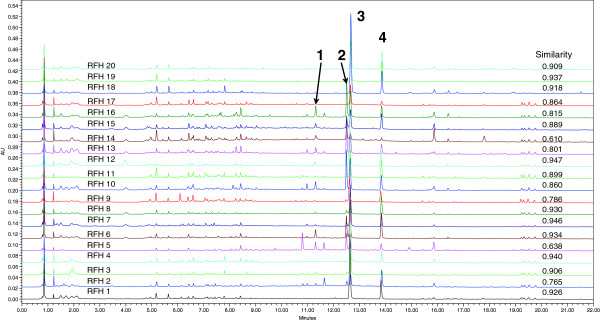
UPLC fingerprinting of twenty RFH samples at 270 nm.

**Figure 3:**
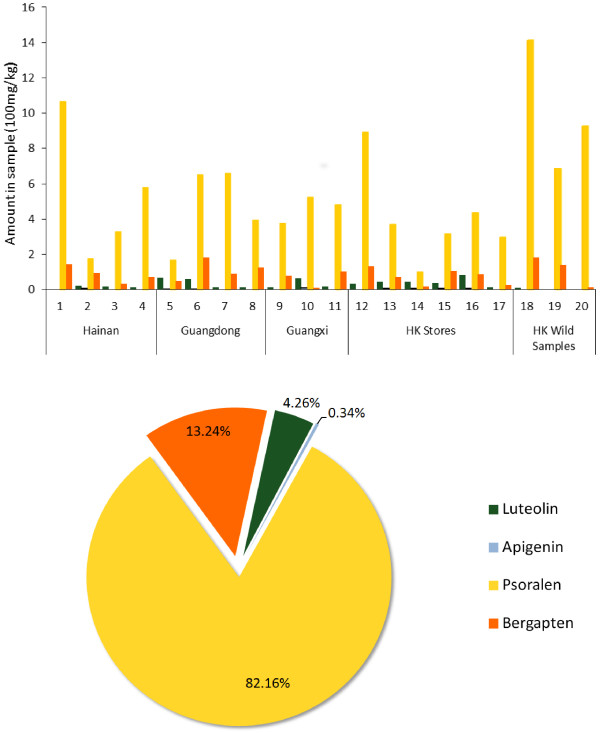
The respective content and average percentage of the four active components in twenty samples.

Twenty RFH samples acquired from five regions were determined using the present method. The fingerprint chromatograms of twenty RFH sample are shown in Figure 2, and the results of quantification are summarized in Figure 3.

As shown in Figure 2, luteolin, apigenin, psoralen and bergapten are the main constituents of RFH. Although the quantified levels varied, the developed analytical procedure was shown to be reproducible and is considered suitable for the analysis of RFH samples.

After calculation (Figure 3), the order of average percentage of the four active components in twenty samples, from most abundant to least abundant, was: psoralen (82.16%) > bergapten (13.24%) > luteolin (4.26%) > apigenin (0.34%). This general ranking was true for all samples. It can be concluded that in the twenty RFH samples, the two coumarins (namely, psoralen and bergapten) were at higher content level than the two flavonoids (luteolin, apigenin). This result is in line with the characteristics of the Moraceae plant in the accumulation of chemical constituents [21,22]. Findings of this study re-confirm that RFH is a popular food with promising potential for further development in commercial products [23,24]. Furthermore, the predominance of psoralen among the four active constituents studied verifies our previous report that this chemical may be responsible for RFH’s primary health benefit [7,8].

### Evaluation of geographical origin

Chromatographic fingerprint similarity evaluation and cluster analysis were used to assess geographical origin of RFH. The chromatographic data were imported into the “Similarity Evaluation System for Chromatographic Fingerprint” software (version 2004 A). The standard fingerprint was generated from the average chromatogram of twenty RFH samples, and then used for similarity evaluation of entire samples [25,26]. In comparison with the standard fingerprint, all RFH samples showed a similarity of at least ≥ 0.8 except the sample RFH 2, 5, 9 and 14 (Figure 2), for their similarity values ranged from 0.61 to 0.79 (Figure 2). Although the peak intensities in the individual chromatograms varied, there was no obvious regular pattern in the global fingerprint in the chosen sources of RFH.

**Figure 4:**
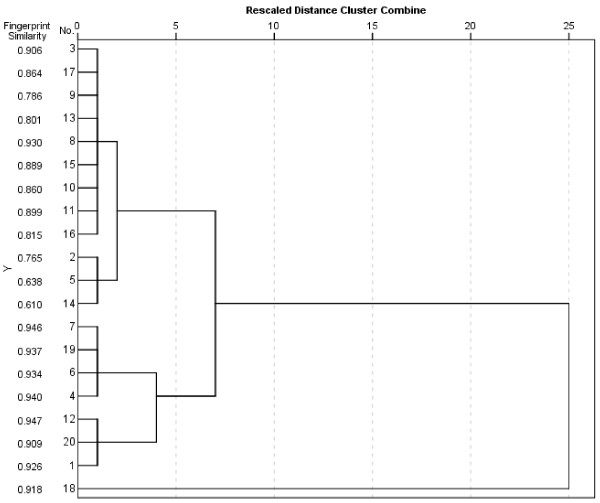
Dendrogram of twenty RFH samples using cluster analysis.

To further verify this result, a cluster analysis was employed here to compare RFH samples from different origins. The hierarchical clustering using classify analysis was performed by SPSS 20.0 software. Between group average linkage method was applied, and rescaled distance was selected as measurement. A dendrogram was resulted from the four component contents of the tested samples. From the results of cluster analysis combined with fingerprint similarity (Figure 4), it was shown that most of RFH samples were clustered within a category at the critical value of 7.5. Sample RFH 18 was distributed outside the category due to its very high content of psoralen. In general, this finding accorded with the similarity evaluation results, which revealed a high level of similarity for RFH samples from different origins.

When the critical value was less than 7.5, the RFH samples were divided two categories. The first category included RFH 3, 17, 9, 13, 8, 15, 10, 11, 16, 2, 5 and 14. The fingerprint similarity values for the most samples (RFH 3, 17, 13, 8, 15, 10, 11 and 16) of the first category ranged from 0.82 to 0.93, and we found these samples contained a moderate content of active components referring to Figure 3. Samples of RFH 2, 5 and 14 in the first category exhibited a similarity from 0.61 to 0.77, and they contained a low content of active components. Thus, the three samples were re-clustered.

The second category included RFH 7, 19, 6, 4, 12, 20 and 1, when the critical value was less than 7.5. The fingerprint similarity values for the samples of this category ranged from 0.91 to 0.95, and we found these samples contained a high content of active components referring to Figure 3. The cluster results also agreed with the findings of fingerprint similarity evaluation, which means global profiles were highly similar for RFH samples from different origins even though their contents of the active components were various.

### Antioxidant assay of the active components

**Figure 5:**
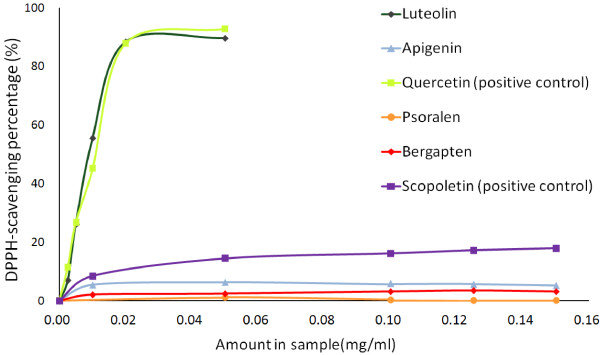
DPPH radical scavenging profiles of the active components.

The antioxidant capacities of the four active components in RFH were measured using DPPH assay. Quercetin and scopoletin served as positive control references for the flavonoids (luteolin and quercetin) and coumarins (psoralen and bergapten), respectively. The results (Figure 5) revealed that the antioxidant potencies of the four active components in descending in order was luteolin > apigenin > bergapten > psoralen, and that the flavonoids exhibited more potency than the coumarins. From the results three findings emerge.

Firstly, the antioxidant potencies of luteolin and quercetin are comparable. According to the obtained profiles, the IC_50_ of luteolin and quercetin were 9.52 μg/mL and 10.08 μg/mL, respectively. About 90% of radical inhibition was reached when their concentrations was 0.02 mg/mL for both quercetin and luteolin.

**Figure 6:**
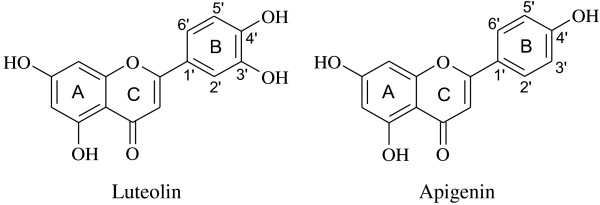
The chemical structures of luteolin and apigenin for structure-activity relationship analysis.

Secondly, in contrast, the antioxidant potencies of apigenin and luteolin, the flavoinoids, varied greatly due to their structure-activity relationship (Figure 6). In the range of 0.01-0.15 mg/mL, the DPPH radical scavenging percentage of apigenin was low (5.26-5.91%), and no significant dose-effect relationship was observed. At the same concentration of 0.02 mg/mL, the scavenging ability of luteolin was found to be 9.40 times of that of apigenin. According to the literature [27,28], the number of hydroxyl group in ring B contributes greatly to the antioxidant potencies of flavonoids. The ring B of luteolin consists of 3′, 4′-dihydroxyl groups, while that of apigenin consists of only one 4′-hydroxyl group, thus luteolin is more easily oxidized and, consequently it appears, exhibits a higher radical scavenging ability than apigenin. The great activity gap between luteolin and apigenin found in our study suggests that the 3′-hydroxyl group is especially essential for the antioxidant potency of such components, and its *o*-dihydroxy groups have better electron-donating properties to form ketones after scavenging radicals this result is in line with the report [29,30].

Thirdly, psoralen and bergapten possessed little effect against free radical reactions, but they are abundant. From the results of chemical quantification (Figure 3), the average proportions of psoralen (82.16%) and bergapten (13.24%) are approximately 20 times of those of luteolin (4.26%) and apigenin (0.34%) in content. Thus, the contribution of psoralen and bergapten cannot be ignored, when the antioxidant effect of RFH is considered.

## Conclusion

The present UPLC-PAD-MS detection proved to be a highly precise and accurate method for quantitative analysis of active components in RFH obtained from different regions. The procedure of DPPH radical scavenging assay adopted for the antioxidant evaluation of the four active components is efficient and reliable. Among the four active components, the coumarin psoralen is most abundant, while the flavonoid luteolin has the strongest antioxidant capacity. Based on the combined results of the chemical quantification and antioxidant assay, it is suggested that both the coumarins and flavonoids are involved in the health benefit of RFH.

## Abbreviations

RFH: Root of *Ficus hirta*; UPLC: Ultra performance liquid chromatography; PAD: Photodiode array detection; DPPH: 2, 2-Diphenyl-1-picrylhydrazyl; ESI: Electrospray ionization; MS: Mass spectrum.

## Competing interests

The authors declare that they have no competing interests.

## Authors’ contributions

HBC initiated and all authors designed the study. The plant samples were collected by LLF, JX and JYZ. The experimental work was carried out by SWS and YLL. The cluster analysis was performed by XCH and YNT using SPSS software. The method developments were conducted by TY, and TY and QLC drafted the manuscript. All authors contributed to data analysis, read and approved the final manuscript.
